# Online support groups for carers of people living with dementia: An investigation of videoconferencing support groups in lockdown

**DOI:** 10.1177/14713012231153431

**Published:** 2023-01-19

**Authors:** Bethany McLoughlin, Helen Atherton, John MacArtney, Jeremy Dale

**Affiliations:** Unit of Academic Primary Care, Warwick Medical School, 2707 University of Warwick, Coventry, UK

**Keywords:** caregivers, peer support, interviews, mixed-methods

## Abstract

**Background:**

This study aimed to explore the experiences of carers of people living with dementia who participated in videoconferencing support groups during the COVID-19 pandemic to investigate their preferences and experiences with online, hybrid, and face-to-face support.

**Methods:**

This convergent mixed methods design study utilised an online questionnaire and semi-structured interviews. Interviews took place over videoconferencing software and were analysed through thematic analysis. Participants were recruited from support groups based in the UK and Ireland.

**Results:**

39 carers of people living with dementia completed the questionnaire and 16 carers participated in interviews. Participants found videoconferencing support groups more convenient, but face-to-face groups more enjoyable. Participants who had found it difficult to access face-to-face groups prior to COVID-19 expressed more positive perceptions of videoconference-based groups. Many felt that hybrid groups would make it easier for more people to attend. However, some carers described lacking the resources and technological skills to participate in online support groups effectively. Some suggested making IT training available may improve the capacity of carers to access support online.

**Conclusion:**

Videoconferencing support groups can be an appropriate way of supporting carers of people with dementia, especially for those who do not have access to face-to-face support groups. However, face-to-face support remains important to carers and should be made available when it can be implemented safely. Hybrid support groups could allow for increased accessibility while still providing the option of face-to-face contact for those who prefer it or are not adept with technology.

## Background

It is estimated that there are over 900,000 people in the UK providing informal care for someone with dementia (Wittenberg et al., 2019). Peer support groups are an avenue for informal carers to find support and advice for managing their situations, as well as reducing social isolation (Greenwood et al., 2013; Keyes et al., 2016). However, it can be difficult for some people to attend face-to-face peer support groups, for example, due to a lack of access to transport or respite care, lack of appropriate groups nearby, or lack of time due to work or caring responsibilities (Ussher et al., 2008). Online support groups could potentially overcome many of these problems and may be a way of making peer support more accessible for some. There are several different approaches to online support groups, for example Facebook groups, online forums, texting groups over WhatsApp, or group video calls over videoconferencing software such as Zoom. However, there is insufficient evidence to draw conclusions about the effectiveness of online peer support groups or compare their effectiveness to face-to-face groups (Boots et al., 2014; Carter et al., 2020).

From March 2020 to May 2021 support groups in the UK were not permitted to meet face-to-face due to the COVID-19 pandemic (Timeline of UK government coronavirus lockdowns and restrictions, 2021). As a result, groups were either cancelled or moved to an online format. This created a population of carers who have experiences of online and face-to-face support groups, and hence an opportunity to learn from their experiences with and preferences about these two methods of participating in support groups.

It has been demonstrated that in workplace settings the use of videoconferencing software during the pandemic substantially increased interest in hybrid working (Gifford, 2022; Phillips, 2020). The extent to which the same applies to support groups is unknown. It is possible that there could be demand for hybrid support groups (whereby a group meets face-to-face with an option to join the meeting remotely through videoconferencing software) or a mixed approach to support groups (where groups use any combination of online and face-to-face meetings e.g., a support group that holds face-to-face sessions and videoconferencing sessions on alternate weeks, or a group that typically meets online but meets face-to-face for special events). Therefore, this research also aimed to investigate carers’ views about using online, hybrid, or a mixed approach for their support group after lockdown restrictions are removed. Its aims were to:

(1) Explore the experiences of carers whose support group moved online during COVID-19.
(2) Investigate carer’s preferences about participating in online or face-to-face support group sessions.
(3) Identify factors affecting carers’ capacity to access online, hybrid, or mixed support in the future.

## Methods

### Study Design

This study used a convergent mixed methods approach utilising a questionnaire and semi-structured interviews (Creswell & Plano Clark, 2011). We aimed to capture preferences about participating in online versus face-to-face support groups using the questionnaire, and to explore the reasons behind these preferences through semi-structured interviews to gain a richer understanding of the needs and experiences of carers of people living with dementia, along with identifying what issues affected access to online support.

### Participants and Recruitment

Participants were required to be over 18 years old, be providing non-professional care for someone with any type of dementia, living in the UK or Ireland, and have regularly attended a face-to-face peer support group that had moved online due to COVID-19 lockdown restrictions. Around 120 organisations that offer support groups for carers of people living with dementia were identified and contacted about the study. Organisations that replied were given details about the study to share with their support group members via email newsletters or at sessions. Interested eligible individuals were directed to a university webpage containing information about the study, a participant information sheet, and a link to the questionnaire. In addition, the opportunity to participate in the study was publicised by Join Dementia Research (Join Dementia Research, n.d.), and interested eligible individuals were directed to the study webpage.

On completing the questionnaire, participants were invited to express interest in an optional interview to discuss their views. All those who did so were contacted by email and provided with an information leaflet and consent form.

### Study Materials and Data Collection

The questionnaire and interview guide were designed to address the two aims of the study. Two carers of people living with dementia were consulted as Patient and Public Involvement (PPI) representatives to ensure that the questionnaire, interview guides, and corresponding participant information sheets were easy to understand, had sensitive and appropriate language, and were manageable in length.

The online questionnaire took approximately 10 min to complete and consisted of closed questions about their experiences at face-to-face and online support groups and their preferences between the two delivery methods. Data was also collected about the participant’s demographic status, and the type of support group that they attended by asking about what happened at a typical session (e.g., singing, talks from experts, dementia café etc.).

Interviews lasted between 45 and 60 min on Microsoft Teams and followed a semi-structured interview topic guide (see supplementary files). The focus of the interviews was to elicit a more in-depth discussion of the participant’s experiences with peer support groups, how the pandemic affected them as a carer, and what support they would like to receive in the future. Interviews were audio and video recorded and were later transcribed verbatim. Identifiable information was pseudo-anonymised. All interviews and transcriptions were carried out by the first author (a female PhD student with training in qualitative interview techniques) and no participants had met the interviewer previously.

The questionnaire was open from 11th March 2021 until 30th April 2021. All interviews took place during this period. Online and postal versions of the questionnaire were made available, but all responses were received via the online questionnaire.

### Data Analysis

#### Questionnaire Data

Frequency of each response was calculated and reported in Table 1. The number of responses meant that only descriptive statistical analysis was suitable.

**Table 1.** Questionnaire participant demographic data.

Characteristic	N	%
Gender		
Female	31	79.5
Male	8	20.5
Age		
18–30	0	0.0
31–40	3	7.7
41–50	3	7.7
51–60	6	15.4
61–70	13	33.3
71–80	10	25.6
81+	4	10.3
Ethnic background		
White	39	100
Sexual orientation		
Heterosexual	38	97.4
Homosexual/lesbian	1	2.6
Employment status		
Employed full time	8	20.5
Employed part-time	6	15.4
Student	0	0.0
Unemployed	1	2.6
Retired	24	61.5
Relationship with care recipient		
Spouse/partner	24	61.5
Parent	13	33.3
Friend/neighbour	2	5.1

#### Interview Data

Thematic analysis was used to identify themes in the data, following the guidelines of Braun and Clarke (Braun & Clarke, 2006). Transcripts were read for familiarisation, and then line by line analysis coding of each transcript was completed with NVivo 12 by one researcher. This was an iterative process, with the researcher re-listening to interview recordings and re-coding throughout analysis. Codes were then grouped into themes based on similarity. Themes were then shared and discussed with co-authors to revise and define the themes. Analysis continued throughout the process of drafting and writing-up, as we returned to the literature to help interpret the findings.

### Ethics

This study received ethics approval from the University of Warwick Biomedical and Scientific Ethics Committee (reference: BSREC 35/20-21). Written consent was received from all interview participants, interview participants consented to their verbatim quotes being published anonymously.

## Results

### Questionnaire Findings

#### Participant Characteristics

In total, 39 carers completed the questionnaire. All participants were white, and the majority identified as heterosexual (38, 97%) and female (31, 79%). Table 1 provides further demographic details. Approximately a third of participants were recruited via independent support groups and two-thirds were recruited from Join Dementia Research.

#### Support Group Demographics

##### Face-to-Face Support Groups

Most participants described that their groups met once a week (n = 20, 51.3%) or once a month (14, 35.9%) with some meeting more than once a week (3, 7.7%) or every other week (2, 5.1%). Participants described that their support group sessions were attended by an average of 16.7 members with a range of 6–85 people.

##### Online Support Groups

The majority of groups met over videoconferencing software, such as Zoom, with one group using WhatsApp calls. Most groups met once a week (24, 61.5%) or once a month (11, 28.2%); three met every other week (3, 7.7%), and one met at irregular intervals(1, 2.6%). Participants described that their online support group sessions were attended by an average of 10.8 members with a range of 3–26 people.

#### Views About Technology

Ten (25.6%) participants reported that they had to buy or learn new technology to attend the support group once it ceased meeting face-to-face, but only two (5.1%) participants felt discouraged from joining a support because of their technology skills. Eight (20.5%) participants reported that they had experienced technical difficulties that had impacted their enjoyment of their online support group sessions.

#### Preferences About Face-to-Face versus Online Support Groups

**Figure 1.**
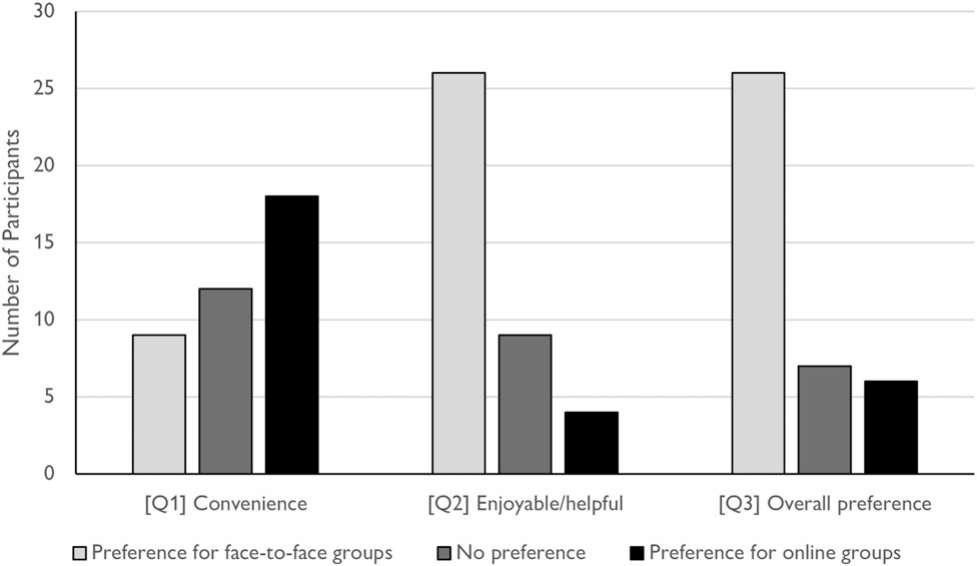
Participant preferences about face-to-face versus online support groups based on questionnaire data.

Participants reported that they found online groups more convenient but found face-to-face groups more enjoyable and helpful. They had an overall preference for face-to-face groups (see Figure 1). Twenty-one (53.8%) reported that they missed around the same number of face-to-face and online sessions, twelve (30.8%) reported that they missed more face-to-face sessions, and six (15.4%) participants missed online sessions more frequently. Four (10.3%) participants said that they would only be interested in attending online sessions from now on, while 26 (66.6%) expressed a preference for attending a mixture of face-to-face and online sessions once the pandemic is over; eight (20.5%) wanted to return to face-to-face sessions.

### Interview Findings

#### Participant Characteristics

All 19 participants who volunteered for interviews were contacted by email, of these a convenience sample of 16 carers (12 female, 4 male) participated in interviews. Thematic analysis identified two main themes: (1) Perceptions of online support groups, and (2) Preferences for future support.

### Theme 1: Perceptions of Online Support Groups

This theme explores the participant’s perceptions of online support groups based on their experiences during the pandemic. None of the participants described having tried videoconferencing support groups prior to lockdown. Their motivation to try utilising online support reflected the disruption or loss of access to support services, social isolation during lockdown, and fears about contracting COVID-19 from face-to-face contact with people outside of their household.

Participants often described a learning curve involved with taking part in a videoconferencing support group which made participation difficult at first, but many participants were able to overcome this and came to appreciate the convenience of taking part in a support group online. However, some participants felt that online support groups failed to replicate the ‘personal touch’ that face-to-face support groups can offer. For participants who attended support groups with the person they care for, there was a varying degree of satisfaction based on how well their care recipient could engage with the sessions. Each sub-theme will now be discussed.

#### Learning Curve

As the majority of participants in the study were unfamiliar with videoconferencing, there was a learning curve for them and the other members of their support group when their support group first moved online. Initially, many participants struggled with developing or understanding the protocol of a videoconferencing meeting (e.g., putting your hand up when you want to speak, muting yourself when not speaking, what to do if your computer freezes etc.).

‘*I’ve enjoyed all the online things. It's been good… once we got going, once we were all bit more confident using technology and things! You know, like learning how to mute yourself.’ (Participant 15)*

‘*[There’s no] protocol. We’ve been thrown into the world of Zoom and there's no sort of rules about it! There's a hand here [on the screen UI], which I presume I raise my hand if there's a group of people, but that would have that would have to be established to start with, whereas most of us just muddle into it without realising there's a hand there.’ (Participant 4)*

‘*I guess the difficulty [with Zoom] is that only one person can speak, and we had a few issues early on about people not understanding that they had to mute themselves so the phone would ring and, you know, they'll be talking over the phone we’d hear everything that they said.’ (Participant 14)*

For most participants, over time they gained confidence in using videoconferencing software. However, some participants never adapted to using videoconferencing, and found that distressing. Though the pandemic motivated them to try online support groups, some had stopped regularly attending their online support groups because they found it too difficult.

‘*It's like life is stressful anyway and then when you're being asked to sort of learn something new, it's like “oh no, can I be bothered?” and it's stopped me from joining all these other [online] groups which could maybe help with things.’ (Participant 11)*

‘*I’m sort of crossing [the online activities I tried] off my list. I couldn't get to grips with it… it’s just alien. It's just too big a jump.’ (Participant 1)*

Finally, some participants were disappointed that other members of their support group did not join in with the online version of the support group or dropped out of it, as having fewer people attend made their experience less useful. Some were also concerned about the wellbeing of those that did not join and were worried that they were lonely or felt abandoned.

‘*The other people in our group were quite a bit older, and whereas our group was maybe 8-9 people sometimes more [when meeting face-to-face], there's never been more than three of us on the Zoom calls. It’s a very, very small number and then you don't benefit so much from the shared experience because each of us are kind of saying the same things over and over again. And so, I mean it does work, but it's not as beneficial. And also, for those people that for whatever reason aren't comfortable using it, they're missing out completely.’ (Participant 13)*

‘*I would go back to the face-to-face group [after lockdown ends], unless it was only available online, just because I think more people would participate. So, you get the benefit of more people joining in. (Participant 14)*

#### The Accessibility and Convenience of Online Meetings

The main positive perception of online support groups was their accessibility and convenience. Prior to lockdown many carers found it difficult to access respite care while they attended a support group face-to-face, or if they attended a joint support group, they found it difficult to get the person they care for out of the house. Some participants were concerned that they may struggle even more to get the person that they care for back to a face-to-face support group after lockdown ends due to a deterioration in mobility and/or progression in dementia severity. For these reasons, online support group meetings may be more accessible for some carers, as it could eliminate the need for respite care.

‘*We're at the stage in our house now where the dementia is so advanced that he can't be left alone. So, I'm constrained by times when carers are here with him. And then because I have a set time, I've got to be back at one because they're going on to another client, I can't be late back. You know, so the constraints are not good for meeting face-to-face.’ (Participant 15)*

‘*Getting [my husband] in the car [to go to a support group meeting] isn't easy. Something that's got a lot more difficult since last March [i.e., when lockdown began] is just getting into the car because we don't use the car very much because we don't go anywhere. He has less opportunity, I suppose, to get used to it, to practice it.’ (Participant 6)*

‘*[My husband] suffers with really severe anxiety attacks about getting in a car. So, a journey that could have taken half an hour, it took us three hours to actually get him there […] Now, if you were in a situation where I was stuck at home and because he wasn't wanting to go [to the face-to-face support group], then [joining a Zoom call from home] it would be a helpful way of me still being involved. That’s the positive side of Zoom.’ (Participant 11)*

Those who were adept with technology, had little free time, or lived far away from their support group saw the greatest benefit of attending a support group online and found it more convenient than attending face-to-face meetings.

‘*[The online support group] was quite convenient because I work part time so I could just fit my work around it, and it wasn't a problem for me. Whereas the face to face, I have to literally schedule it in and drive over there, and so it takes a bit longer.’ (Participant 13)*

‘*For the face-to-face meetings, it was half an hour away from my house and I'd have to get somebody to look after my mum for two to three hours. So, I didn't have to do that, you know, it was just so handy and so convenient to do it online. And it saves time! This is much more efficient!’ (Participant 7)*

‘*A lot of carers if they're looking after somebody in their own home, they can't leave that home to travel [into town] for a meeting, even though they have a lot of value to add. So, I think participation in a conference via a zoom link or a Microsoft Teams link will continue. I think the world of extended reach through communication will not go away […] and for people who can't get out very often, it's a lifeline. It really is.’ (Participant 12)*

#### The Personal Touch

Some participants felt that the protocol of using videoconferencing was awkward or difficult to navigate, making conversations feel ‘cold and impersonal’. This was particularly striking when combined with problems with IT, such as having an unreliable WiFi connection.

‘*I'd say it's just more stilted on Zoom, you just waiting for your chance to speak, and you don't bounce off each other the same. It just doesn't happen on a Zoom. It feels more formal.’ (Participant 11)*

‘*The cons of it are it's the thing of technology. There's freezing, going on your reception goes and you have to come back again. There's a time lag that I find really difficult and then often I’m somehow butting into a conversation when I don't mean to.’ (Participant 4)*

‘*These kinds of meetings online, they feel a bit more like a work environment, a team meeting. They’re quite tiring, even for me. […] I prefer face-to-face, it's more natural, it's warm, sociable. You connect with people more when you’re face-to-face.’ (Participant 7)*

Some participants commented that online support group meetings were missing ‘the personal touch’ as they do not facilitate smaller moments of connection that can only happen face-to-face, for example, hugs or eating and drinking together.

‘*It's those separate little conversations that you're having as you're going to make yourself a cup of coffee that helps builds up the friendship and the relationship and becomes a support.’ (Participant 5)*

‘*Face-to-face if somebody is upset, and you know there are occasions when people do get really upset, you know you can always go give somebody a hug if they need it. When you're on screen you can't. It's not so personal.’ (Participant 8)*

‘*I think face-to-face, you get a more human element out of it. Every time I go, I cook cakes and I take cakes or biscuits to the group, and I make it sort of special. It's like a special thing, […] it's about the personal touch. (Participant 14)*

#### Care Recipient Engagement with videoconferencing

For participants who attended online support groups alongside the person they care for, their capacity to engage with the videoconferencing support group impacted the carer’s perception of online support groups. For those with care recipients who were able to engage well, the sessions were seen as helpful and as an opportunity to spend some enjoyable time together.

‘*[The online support group meets] every other week and I notice a difference. He actually smiles a lot. It's just that's a bit of stimulation for him because they're so good at bringing things out of, you know, people with dementia. You know, by chatting and being able to say the right things. That's good.’ (Participant 8)*

‘*So, we started doing the zoom choir and she loves it, said she absolutely loves it. You know, she loves that she can see her friends and she can wave to them. Mum just gets stuck in, and she's just... she's our mum again for that time. We're not really performing a caring role at that point; we're just spending quality time with our mum.’ (Participant 3)*

‘*When he responds [and engages in the online support group] it really pleases me, it really does. It's not very often I hear him laugh and I think that it’s lovely to hear him laugh. (Participant 6)*

‘*I know certain people who aren't able to do [the online support group] because the people they care for don't engage with the screen particularly well. They find it confusing, whereas with my mum there hasn't been a problem in her engagement with it. So, it's been marvellous. She behaves as if she's got a real person in front of her.’ (Participant 16)*

However, many participants reported that the person they cared for was not able to engage with the online support group, which gave them a negative perception of online support groups. Participants spoke about how their care recipient’s age and/or dementia made it difficult for them to understand technology, or the social rules of videoconferencing.

‘*[My wife] couldn’t understand the screen with the dementia. She couldn't even understand that her daughter was on Zoom. It didn't mean anything on the little screen, forget it! Our age group weren’t brought up with screens and that. I found it difficult with Zoom to feel that I'm actually in a meeting anyway. […] I just think that what was on offer before the pandemic was useful, very useful. What’s on offer since the pandemic is totally useless [for my wife].’ (Participant 1)*

‘*[The person I care for] doesn’t get it when you've got to wait. You've got a group of people, so you've got to wait for somebody to stop talking before you can. So, he’ll just like talk over people and he doesn't realize he's doing it. […] He wouldn't be able to access Zoom without me.’ (Participant 11)*

### Theme 2: Preferences for Future Support

Participants were asked about what type of support they would like to access in the future, and whether they would be interested in attending online or mixed support groups in the future.

#### Interest in Hybrid and Mixed Groups

Participants who expressed a desire for a mixture of face-to-face and online support saw online support as a way to supplement face-to-face support at times when they could not attend in person and/or a way to help other group members to stay involved.

‘*I think the mix and match is here to stay. […] I think online has got its benefits, especially for those people maybe who have a disability and who can't get to a group. I'd be quite happy to keep it online in that regard.’ (Participant 14)*

‘*Moving forward, I think it's getting the balance between being able to get together on a regular basis in a live situation plus having Zoom as a backup whenever it's needed. I'm not going to stop doing Zoom sessions when everyone is able to get back together again. If things are still available online, I'll still be accessing them as well.’ (Participant 16)*

‘*For me I like face-to-face. And for the group that I'm in I think they all work better with face-to-face. I think the Zoom calls we've had have been a good alternative and I think if there was a way of being able to incorporate both online access and face-to-face, for those who can't make a face-to-face meeting then that would be a good thing.’ (Participant 13)*

A hybrid support group model, whereby the group meets face-to-face but there is an option to join remotely through videoconferencing was seen as a viable option.

‘*For the future if I can't get him out the house then that would be a social event taken away from me as well. Whereas if there was that option to dial into others that were having the meeting face-to-face then, I can see that as a positive. I’d still be looking at those meetings for support and a laugh and a bit of interaction with others.’ (Participant 10)*

‘*How could we have got through lockdown without [Zoom]? It’s not my ideal, but it's been really, really useful and a life saver. I'd still rather be there online [in a hybrid setting] than not be there, obviously.’ (Participant 4)*

Participants that had struggled with technology expressed that training would be appreciated and may help them access online support in the future and take advantage of a mixed/hybrid group situation.

‘*When things open up again, I think it would be quite good if there was sort of a basic place that you could go and get some tuition or a class [about using the internet]. I'd be quite willing to do that because I really feel so old fashioned now. I don't understand all the terminology and it's ridiculous.’ (Participant 8)*

‘*I would have loved to have done training [before lockdown] and the one thing I feel is missing for my age group is training on computers. […] I think that would be lovely, it really would. And then we won’t shy away from technology so much.’ (Participant 4)*

#### Interest in Face-to-Face or Online Support Only

However, some participants who had experienced significant difficulties using technology but had previously been able to easily travel to face-to-face groups were uninterested in support groups continuing online. They felt that online support groups could not replace face-to-face interactions.

‘*I think that being out of the house that was my escape. […] Sometimes you feel your head is bursting, you just need the calm and it's nice to get away. The carers group face-to-face is that place. (Participant 8)*

‘*It’s driving 15 minutes up the road, that far outweighs the unnaturalness of being on the phone like this… I would definitely not choose Zoom and I can’t wait for the Zoom to be over, and we can actually meet up with people face-to-face. Get back to having laughs and jokes around the table and eating a meal with them.’ (Participant 11)*

However, returning to a purely face-to-face delivery model could be a barrier that prevents vulnerable people from attending support groups. One participant who really struggled to attend support groups face-to-face because there were no groups nearby was concerned that the online support that had been accessing would end after lockdown due to a lack of interest from others in their group.

‘*People are still waiting to meet face-to-face. And [after lockdown ends] they might sort of say it's not worth doing it online anymore, and then that will stop. And then I've lost that contact with people.’ (Participant 15)*

‘*I just think the more you can involve people and the wider you can have that audience the better because you know there is a real shortage of [support] groups [for carers]. And yeah, we haven’t got resources for a lot of [support] groups, so if you can spread that resource [by using the internet] and make it more easily accessible to people, that can only be a good thing.’ (Participant 13)*

## Discussion

This study sought to explore the experiences of carers of people living with dementia accessing online support groups during the first 15 months of the COVID-19 pandemic, and their preferences about future participation in online and face-to-face support groups. Most participants had found videoconferencing support groups were beneficial and convenient, which is consistent with other findings in the literature (Kovaleva et al., 2019; Marziali & Garcia, 2011; Masoud et al., 2021; Wallace et al., 2021). Though participants found online support more convenient, they considered face-to-face groups to be more enjoyable and more suited to offering emotional and social support. Survey results found that most participants preferred face-to-face groups overall.

Some carers found it much more difficult to attend face-to-face support groups than online support groups, for reasons such as a lack of respite care, difficulties with getting the person they care for to the support group, or there being no groups nearby or at appropriate times. Similar barriers to accessing support groups have been reported previously in the literature (Ussher et al., 2008). For these carers, online support groups were more accessible, and there were concerns expressed about the loss of such accessibility if support groups returned to solely meeting face-to-face. Conversely, those who were not adept with technology struggled to participate in online support groups during lockdown, and some participants suggested that fewer people attended online support groups due to lack of access to technology or lack of confidence using technology. The questionnaire responses also appeared to indicate that online groups had fewer members which may suggest that members who had previously attended face-to-face groups stopped attending when the group moved online. It is possible that a substantial proportion of the population of carers of people living with dementia may have difficulties with accessing online support. Using the internet to communicate has been shown to be associated with higher quality of life, and lower levels of depression and social isolation in middle-aged and older adults (Stockwell et al., 2021; Wallinheimo & Evans, 2021; Yu et al., 2021). Therefore, providing clearer guidance and training with technology to carers to help them access online support in the future should be a priority.

Additionally, some participants reported that their care recipients had difficulty engaging with online support. Some felt that this loss of contact had contributed to a deterioration in their care recipient’s mental health, a finding that has been reported elsewhere in the literature (Barguilla et al., 2020; Tsapanou et al., 2021; Van Maurik et al., 2020). Findings about the suitability of videoconferencing for people living with dementia reported in the literature are variable, and it appears that the acceptability of videoconferencing differs between individuals according to the type and severity of dementia (Agustus et al., 2021; Cullum et al., 2006; Vellani et al., 2022). Further research is needed to investigate how to improve the accessibility of videoconferencing for people living with dementia.

Two-thirds of survey respondents expressed a preference for a mixture of online and face-to-face support in the future. Interview participants recognised that online support groups’ convenience and having a mixture of online and face-to-face support or using a hybrid system would enable more carers to attend than a group that was solely face-to-face. How this interest in online/mixed/hybrid support groups continues as COVID-19 safety concerns diminish or if this interest will translate in a permanent change in the provision of support groups needs further investigation. In particular, it is unclear whether support groups will have the interest, resources, or expertise to provide both online and face-to-face sessions.

### Limitations

Recruitment was difficult which led to a smaller sample size than was planned. It is likely that this was due to COVID-19, as many organisations said that they were not able to contribute to recruitment due to stretched resources or suggested that their members would be too busy to take part in research at that time. It is also possible that the number of responses was partly a result of many support groups not transitioning their sessions to an online format, meaning that relatively few carers met the eligibility criteria for the study. Therefore, due to the small sample size of this study, the quantitative findings should be interpreted with particular caution.

It should be noted that participants were all white and almost all heterosexual (see Table 1), so their views might not be representative of those from other groups. This could potentially reflect the demographic makeup of the attendees of support groups for people affected by dementia, though the demographic makeup of support groups for this population is not well understood. Investigating the use of online and face-to-face support groups by people from minority groups could be an important target for future research.

Finally, many of the participants struggled with some technology-based aspect of the questionnaire and/or interview (e.g., how to return the signed consent form as a PDF, how to join the Teams meeting). It is possible that the demands of taking part in an interview online could have led to a non-representative sample, as only those who felt confident in using videoconferencing volunteered for an interview.

## Conclusions

This study’s findings indicate that for carers of people living with dementia that experienced attending a videoconferencing support group during the first year of the COVID-19 pandemic, such groups were generally viewed as convenient but lacking the social and emotional support and enjoyment of attending face-to-face support groups. For some carers, lack of experience with technology made attending a support group online difficult or unenjoyable. However, other carers valued the accessibility of online support groups given the difficulties that they had previously experienced in attending face-to-face groups which had sometimes prevented involvement. Overall, this suggests that online support groups are likely to be of importance to some carers beyond the context of the COVID-19 pandemic. Hybrid support groups could allow for increased accessibility while still providing the option of face-to-face contact for those who prefer it or are not adept with technology, but further research is needed to establish how best to run such groups. In addition, providing guidance and training to carers about how to use videoconferencing software and technology in general could help carers access online support groups more successfully in the future.
